# Supplementary material for: Inflammatory cytokines promote interferon regulatory factor (IRF) transcriptional activity in human pulmonary epithelial cells through the induction of IRF1 by nuclear factor-κB
Source: PLoS One. 2025 Dec 8;20(12):e0329244. doi: 10.1371/journal.pone.0329244 (PMC12685198; doi:10.1371/journal.pone.0329244)
Supplement: S1 File — This file contains supplementary figures and legends 1–6, and supplementary tables 1 and 2 which contain the oligonucleotide sequences used in this study. (PDF) [file pone.0329244.s001.pdf]

Journal Title: PLoS One
Manuscript #: PONE-D-25-37763

Supplemental data: "Inflammatory cytokines promote interferon regulatory factor (IRF) transcriptional activity in human pulmonary epithelial cells through the induction of IRF1 by nuclear factor-κB"

Amandah Necker-Brown[1], Mahmoud M. Mostafa[1], Andrei Georgescu[1], Andrew J. Thorne[1], Priyanka Chandramohan[1], Cora Kooi[1,2], Keerthana Kalyanaraman[1], Alex Gao[1], Akanksha Bansal[1], Sarah K. Sasse[3], Anthony N. Gerber[3,4], Richard Leigh[2] and Robert Newton[1*]

Supplementary Figures

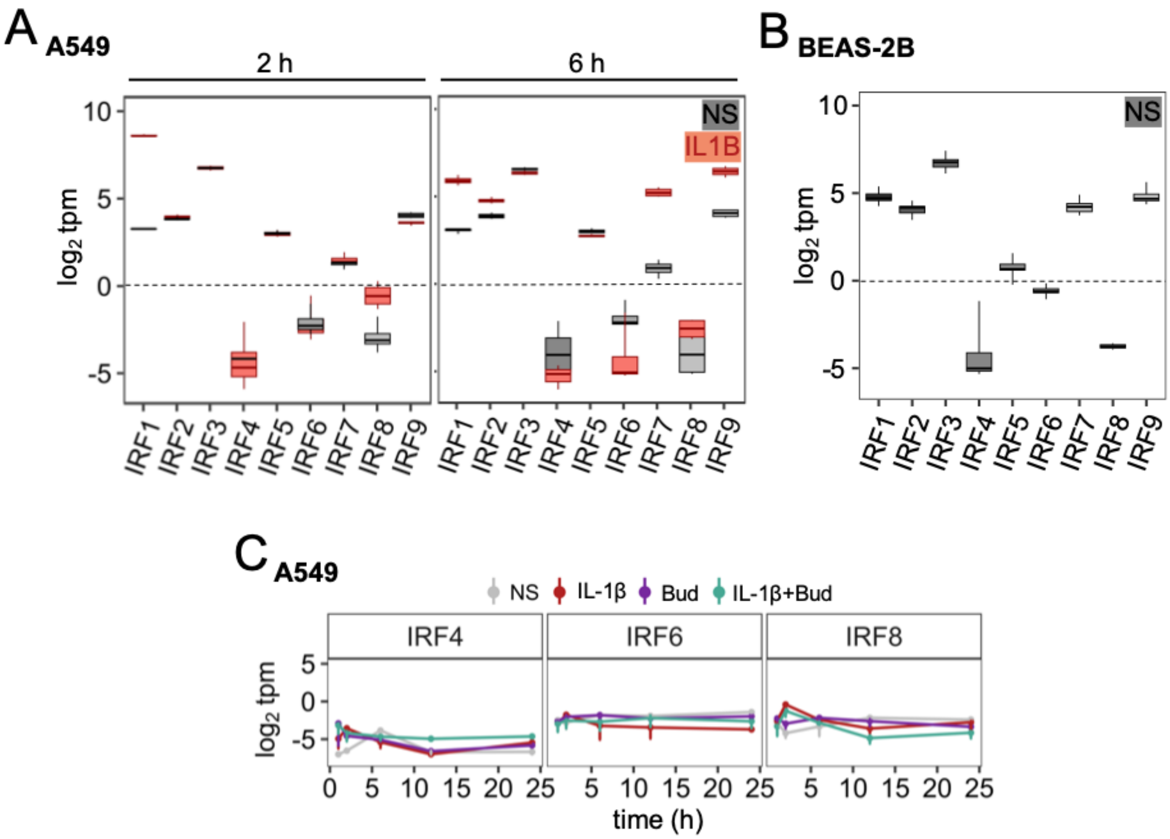

Supplementary Figure 1. IRF expression in A549 and BEAS-2B cells. (**A, C**) A549 or (**B**) BEAS-2B cells were either not stimulated (NS) (**B**) or treated with IL-1β (1 ng/ml) and/or budesonide (300 nM; Bud) for the indicated times (**A, C**). RNA from 4 independent experiments was prepared prior to mRNA sequencing. Data are presented as $\log_2$ transcripts/million (tpm) and are plotted means ± SE or box-and-whisker plots.

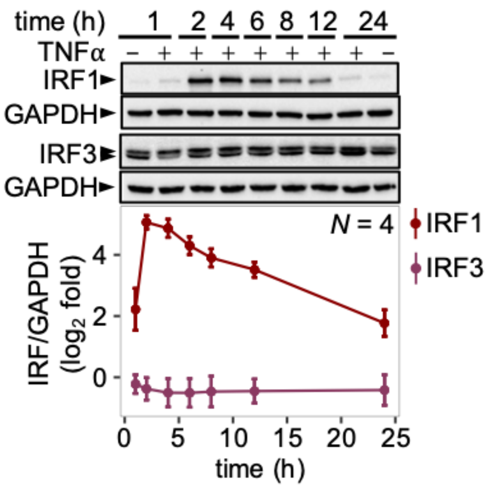

Supplementary Figure 2. IRF1 and IRF3 protein expression in A549 cells following treatment with TNFα. A549 cells were either not stimulated or treated with TNFα (10 ng/ml) for the indicated times prior to harvesting for western blot analysis. Data from *N* independent experiments were expressed as IRF1/GAPDH and are plotted as log_2 fold of no stimulation (t = 0) as means ± SE.

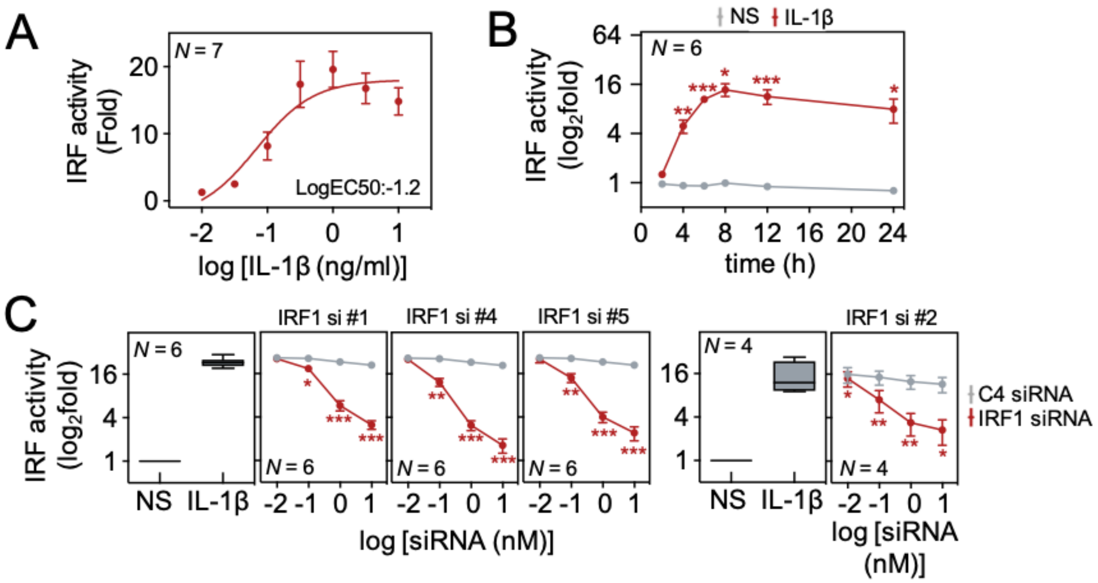

Supplementary Figure 3. Effect of IL1B on activation of IRF1-dependent transcription in A549 cells. (**A**) A549 cells harbouring a stably transfected 6×IRF1 luciferase reporter in (A) were either not stimulated (NS) or treated with 0.003, 0.01, 0.03, 0.1, 0.3, 1, 3 and 10 ng/ml of IL-1β for 8 h (**B**) A549 cells harbouring a stably transfected 6×IRF1 luciferase reporter were treated with IL-1β (1 ng/ml) as indicated. Cells were harvested at the indicated times for luciferase activity determination. (**C**) A549 cells harbouring a stably transfected 6×IRF1 luciferase reporter were treated with increasing concentrations (0.01, 0.1, 1 or 10 nM) of; control siRNA (C4), each of the individual IRF1 siRNAs used in the pool in Figure 2 (#1, 2, 4 or 5 as indicated) prior to either no stimulation of treatment with IL-1β (1 ng/ml). Cells were harvested after 8 h for luciferase activity determination. All data, from *N* experiments, are plotted as fold or $\log_2$fold of no stimulation (NS) ± SE. Using relative light units, significance was tested by one-way ANOVA with Tukey's post-hoc test. * $P \leq 0.05$, ** $P \leq 0.01$ or *** $P \leq 0.001$ indicates significance relative to IL-1β treated cells (C) or non-stimulated cells (B).

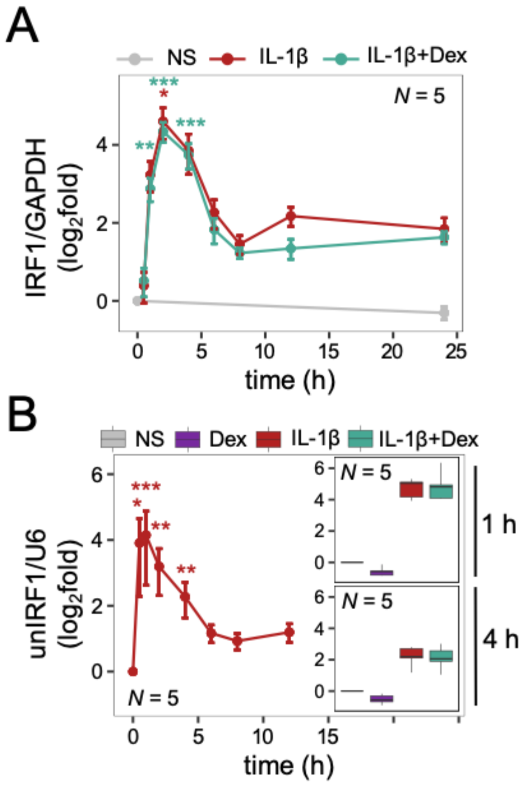

Supplementary Figure 4. Transcriptional activation of IRF1 expression by IL-1β and effect of dexamethasone cotreatment. (**A**) A549 cells were either not stimulated (NS) or treated with IL-1β (1 ng/ml) and/or dexamethasone (1 μM; Dex) for the indicated times prior to harvesting for qPCR analysis of IRF1 and GAPDH mRNA. (**B**) cDNA samples from (A) were analysed by dPCR for unspliced IRF1 (unIRF1) and U6 RNA. Data from *N* independent experiments were normalized to a control gene (GAPDH or U6) and are presented as $\log_2$fold of NS. All data are plotted as means ± SE or box-and-whisker plots. Using fold of NS values, significance was tested by one-way ANOVA with a Tukey's post-hoc, or in B (righthand panels), by paired t test using normalized unIRF1/U6 values comparing IL-1β to IL-1β with Dex * $P \leq 0.05$, ** $P \leq 0.01$, *** $P \leq 0.001$ indicates significance relative to non-stimulated cells.

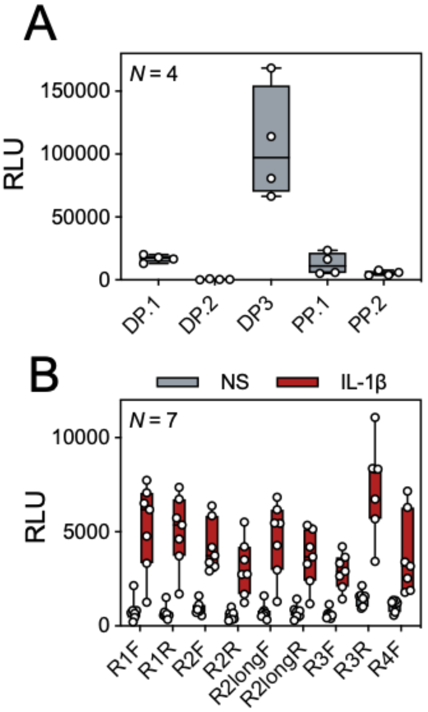

C

	NS			IL-1β		
	mean (fold)	SD	N	mean (fold)	SD	N
R1F	833.7	620.6	7	5267.5	2304.3	7
R1R	704.1	375.0	7	5032.5	1910.8	7
R2F	927.9	347.6	7	4229.7	1361.9	7
R2R	554.5	255.1	7	3097.6	1466.0	7
R2longF	745.6	411.2	7	4642.6	1946.0	7
R2longR	755.6	358.1	7	3600.6	1482.5	7
R3F	626.8	246.5	7	2894.4	947.7	7
R3R	1482.6	402.9	7	7421.6	2425.1	7
R4F	1098.5	315.7	7	3750.0	2133.3	7

Supplementary Figure 5. Basal and IL-1β-stimulated reporter activity of *IRF1* reporter clones. (**A**) A549 cells stably transfected with reporter constructs containing long *IRF1* promoter segments (DP: Distal Promoter, PP: Proximal Promoter) in the forward orientation as depicted in Fig 7D, using the non-stimulated data from Fig 8A. (**B, C**) A549 cells stably transfected with reporter constructs containing short *IRF1* promoter fragments cloned in both forward and reverse orientations. Reporter cell lines were either not stimulated or treated with IL-1β for 6 h prior to luciferase activity determination. Data are from cells that were transfected at the same time and were included in fig 8B. Data from *N* independent experiments were expressed as relative light units (RLU), plotted as box-and-whisker plots, and individual data points are shown. Fold over not stimulated (NS) values listed in (C).

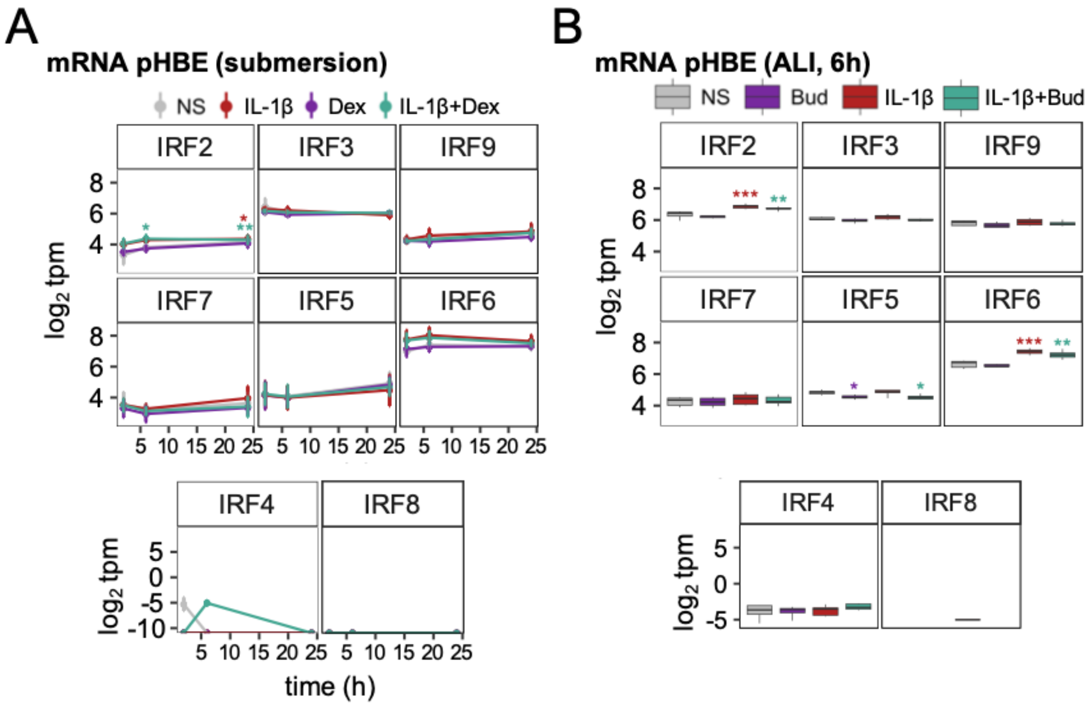

Supplementary Figure 6. IRF mRNA expression in pHBECs in submersion or ALI culture. Primary human bronchial epithelial (pHBE) cells, grown as submersion culture or at air-liquid interface (ALI), were either not stimulated (NS) or treated with IL-1β (1 ng/ml) and/or dexamethasone (1 μM; Dex) or budesonide (300 nM; Bud) for the indicated times. RNA from 4 individuals (pHBE) was prepared prior to RNA sequencing and results are presented as $\log_2$ transcripts/million (tpm). All data are plotted as box-and-whisker plots. In (A), tpm values for each IRF were used for significance testing by one-way ANOVA with Tukey's post-hoc test * $P \leq 0.05$, *** $P \leq 0.001$ indicates significance relative to NS.

Supplementary Tables

Supplementary table 1. qPCR and ChIP primers

Primer name	Method	Sequence (5’ -> 3’)
IRF1 Forward	qPCR	CTCACTGCAGCCCCTGCGTC
IRF1 Reverse	qPCR	TGGGCATGTTGGCTCTGCTGC
GAPDH Forward	qPCR	TTCACCACCATGGAGAAGGC
GAPDH Reverse	qPCR	AGGAGGCATTGCTGATGATCT
MYOD ChIP Forward	ChIP	TGCAGGAGATGAAATACTAAGCAAGTA
MYOD ChIP Reverse	ChIP	AGATTGGAAACTGAGGACTTTAGTTAGAG
MYOG ChIP Forward	ChIP	CCAATGAGACTGAGTGGGTTTTC
MYOG ChIP Reverse	ChIP	TCACCAGAGAAGACTGCTTTGC
OLIG3 ChIP Forward	ChIP	GGCAAGGACAGAGACAATCATA
OLIG3 ChIP Reverse	ChIP	CTCTGTGTTCTCGCTTTGGA
R1 ChIP Forward	ChIP	CTGCAAGAGTGGAGCTTGT
R1 ChIP Reverse	ChIP	GGATTCCCAGGTTGAGTCAC
R2 ChIP Forward	ChIP	TCGGTTTCTTGCAAACGC
R2 ChIP Reverse	ChIP	ACTCCTCGATTTCCCTGTTG
R3 ChIP Forward	ChIP	CCATAATGAGCATCTTTCCATGTC
R3 ChIP Reverse	ChIP	TGGAAATAACCTAACTGTCCTACAA
R4 ChIP Forward	ChIP	CACGTCTTGCCTCGACTAAG
R4 ChIP Reverse	ChIP	GGGAATCCCGCTAAGTGTTT
unIRF1 Forward	dPCR	CCCGGGATTTGGTTGGAATTA
unIRF1 Reverse	dPCR	TCCGCACTGAGTGTAGTACC
unU6 Forward	dPCR	AATTGGAACGATACAGAGAAGATTAGC
unU6 Reverse	dPCR	GGAACGCTTCACGAATTTGC

Supplementary table 2. Cloning primers

Primer name	Method	Sequence (5’ -> 3’)
DP.1 Reverse	Promoter cloning	ATGGTTGGTACCGGACCAGCATCACTGTTTCTA
DP.2 Reverse	Promoter cloning	ATGGTTGGTACCACTCCTCCCTCATTCCCTAAA
DP.3 Reverse	Promoter cloning	ATGGTTGGTACCGTGAGATAATGGGCACAGAGAA

DP all Forward	Promoter cloning	ATGGTTCTCGAGTCCCCTCTCAAAGGTTTGGC
PP.1 Reverse	Promoter cloning	ATGGTTGGTACCACTCCTCCCTCATTCCCTAAA
PP.2 Reverse	Promoter cloning	ATGGTTGGTACCGGACCAGCATCACTGTTTCTA
PP all Forward	Promoter cloning	ATGGTTCTCGAGCTCAAAGGCGTACTCACCTC
R1 Forward (+ orient)	Enhancer cloning	ATGGTTCTCGAGCCTCCCTAGCATCCCTGTAT
R1 Reverse (+ orient)	Enhancer cloning	ATGGTTGGTACCCACTGTCCTGAGCCTCATTT
R2 Forward (+ orient)	Enhancer cloning	ATGGTTCTCGAGTTCGGTTTCTTGCAAACGC
R2 Reverse (+ orient)	Enhancer cloning	ATGGTTGGTACCTGGGCAGGTGCATTCAG
R2long Forward (+ orient)	Enhancer cloning	ATGGTTCTCGAGCGCAGGCACTCAGATGG
R2long Reverse (+ orient)	Enhancer cloning	ATGGTTGGTACCGAGACCTCATCGTATAAGGACAATAA
R3 Forward (+ orient)	Enhancer cloning	ATGGTTCTCGAGCCATAATGAGCATCTTTCCATGTC
R3 Reverse (+ orient)	Enhancer cloning	ATGGTTGGTACCAGTTTCATTGGTAAATCCTAACTGG
R4 Forward (+ orient)	Enhancer cloning	ATGGTTCTCGAGCACGTCTTGCCTCGACTAAG
R4 Reverse (+ orient)	Enhancer cloning	ATGGTTGGTACCCAGCCGCCCTGTACTTC
R1 Forward (- orient)	Enhancer cloning	ATGGTTGGTACCCCTCCCTAGCATCCCTGTAT
R1 Reverse (- orient)	Enhancer cloning	ATGGTTCTCGAGCACTGTCCTGAGCCTCATTT
R2 Forward (- orient)	Enhancer cloning	ATGGTTGGTACCTTCGGTTTCTTGCAAACGC
R2 Reverse (- orient)	Enhancer cloning	ATGGTTCTCGAGTGGGCAGGTGCATTCAG
R2long Forward (- orient)	Enhancer cloning	ATGGTTGGTACCCGCAGGCACTCAGATGG
R2long Reverse (- orient)	Enhancer cloning	TGGTTCTCGAGGAGACCTCATCGTATAAGGACAATAA
R3 Forward (- orient)	Enhancer cloning	ATGGTTGGTACCTCCATAATGAGCATCTTTCCATGTC
R3 Reverse (- orient)	Enhancer cloning	ATGGTTCTCGAGAGTTTCATTGGTAAATCCTAACTGG
R4 Forward (- orient)	Enhancer cloning	ATGGTTGGTACCCACGTCTTGCCTCGACTAAG
R4 Reverse (- orient)	Enhancer cloning	ATGGTTCTCGAGCAGCCGCCCTGTACTTC
S1 mut Forward	Mutagenesis	ACTCCAGGGGCAGCC
S1 mut Reverse	Mutagenesis	AGGTTGAGTCACCAGGG
S2 mut Forward	Mutagenesis	AATACTGCAAGAGTGGAGC
S2 mut Reverse	Mutagenesis	CTGGCCCTCTCCCAG
S6 mut Forward	Mutagenesis	ACCTGAATGCACCTGCC
S6 mut Reverse	Mutagenesis	CGGGGCCGGTTTTCC
S7 mut Forward	Mutagenesis	GGGAACCGAACCACG

S7 mut Reverse	Mutagenesis	CGATTTCCCTGTTGCC
S8 mut Forward	Mutagenesis	CAGTTAGGATTTACCAATGAAAC
S8 mut Reverse	Mutagenesis	CCTAAGCAAAAAATTATTGATATGC
S10 mut Forward	Mutagenesis	CAGCCCTGGCCGGCC
S10 mut Reverse	Mutagenesis	CTAAGTGTTTGGATTGCTCGGTGGC
