# Supplementary material for: Inflammatory cytokines promote interferon regulatory factor (IRF) transcriptional activity in human pulmonary epithelial cells through the induction of IRF1 by nuclear factor-κB
Source: PLoS One. 2025 Dec 8;20(12):e0329244. doi: 10.1371/journal.pone.0329244 (PMC12685198; doi:10.1371/journal.pone.0329244)
Supplement: S2 File — The original, uncropped images for each representative blot are shown with molecular weight marker included. Any lanes not included in the final figure have been marked with an “X” above each lane. (PDF) [file pone.0329244.s002.pdf]

Raw Images

"Inflammatory cytokines promote interferon regulatory factor (IRF) transcriptional activity in human pulmonary epithelial cells through the induction of IRF1 by nuclear factor-κB"

Amandah Necker-Brown[1], Mahmoud M. Mostafa[1], Andrei Georgescu[1], Andrew J. Thorne[1], Priyanka Chandramohan[1], Cora Kooi[1,2], Keerthana Kalyanaraman[1], Alex Gao[1], Akanksha Bansal[1], Sarah K. Sasse[3], Anthony N. Gerber[3,4], Richard Leigh[2] and Robert Newton[1*]

Main figures

Fig 1B

IRF1

Loading order

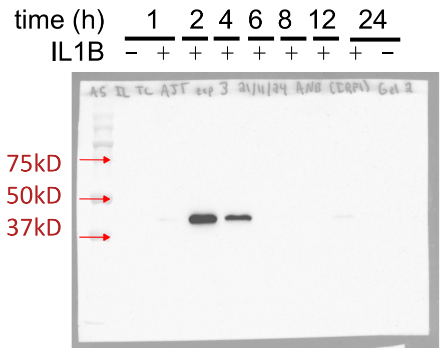

IRF

**Cell line**	**A549**
**Treatments**	IL-1β timecourse
**Experiment # (Person)**	Exp 3 (AJT)
**Date of blot**	21/11/24
**Person running blot**	ANB
**Primary Antibody**	IRF1

GAPDH

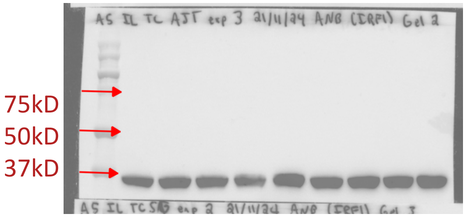

**Cell line**	**A549**
**Treatments**	IL-1β timecourse
**Experiment # (Person)**	Exp 3 (AJT)
**Date of blot**	21/11/24
**Person running blot**	ANB
**Primary Antibody**	GAPDH

IRF2

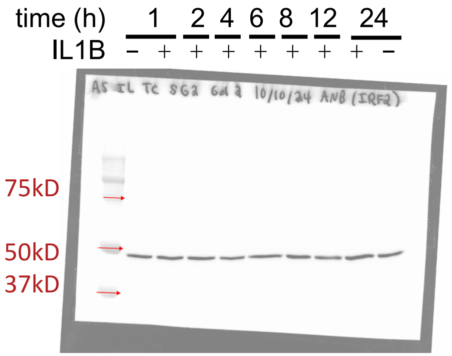

**Cell line**	**A549**
**Treatments**	IL-1β timecourse
**Experiment # (Person)**	Exp 2 (SG)
**Date of blot**	10/10/24
**Person running blot**	ANB
**Primary Antibody**	IRF2

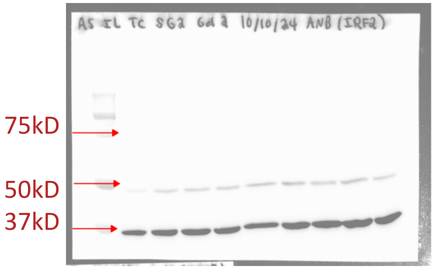

**Cell line**	**A549**
**Treatments**	IL-1β timecourse
**Experiment # (Person)**	Exp 2 (SG)
**Date of blot**	10/10/24
**Person running blot**	ANB
**Primary Antibody**	GAPDH

IRF3

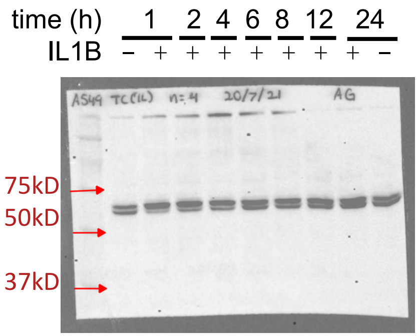

**Cell line**	**A549**
**Treatments**	IL-1β timecourse
**Experiment # (Person)**	Exp 4 (AG)
**Date of blot**	20/07/2021
**Person running blot**	AG
**Primary Antibody**	IRF3

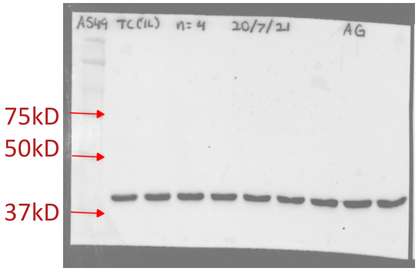

**Cell line**	**A549**
**Treatments**	IL-1β timecourse
**Experiment # (Person)**	Exp 4 (AG)
**Date of blot**	21/07/2021
**Person running blot**	AG
**Primary Antibody**	GAPDH

IRF9

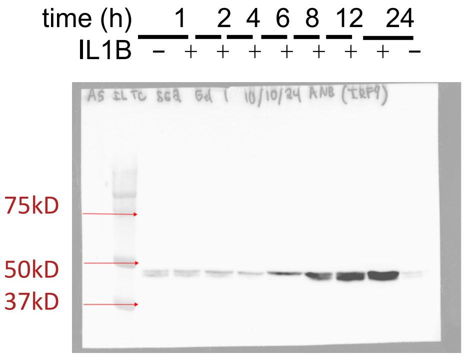

**Cell line**	**A549**
**Treatments**	IL-1β timecourse
**Experiment # (Person)**	Exp 2 (SG)
**Date of blot**	10/10/24
**Person running blot**	ANB
**Primary Antibody**	IRF9

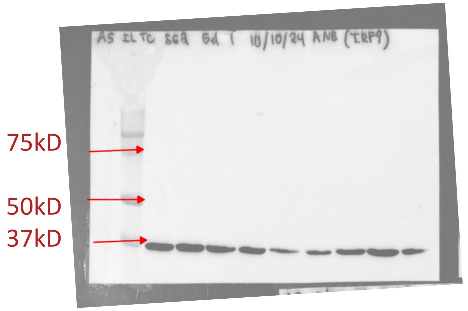

**Cell line**	**A549**
**Treatments**	IL-1β timecourse
**Experiment # (Person)**	Exp 2 (SG)
**Date of blot**	10/10/24
**Person running blot**	ANB
**Primary Antibody**	GAPDH

Fig 2A

	IRF1	IRF2	IRF3	IRF9

Loading order

IRF1 siRNA
C4 siRNA
lipid + +
IL-1β 2h – – + + + + + + + + + +

IRF2 siRNA
C4 siRNA
lipid + +

IRF3 siRNA
C4 siRNA
lipid + +

IRF9 siRNA
C4 siRNA
lipid + +
IL-1β 24h – – + + + + + + + + + +

IRF

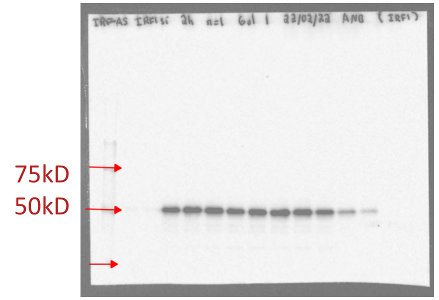

Cell line	A549
Treatments	IRF1 siRNA 2h IL1B
Experiment # (Person)	Exp #1 (ANB)
Date of blot	22/02/2022
Person running blot	ANB
Primary Antibody	IRF1

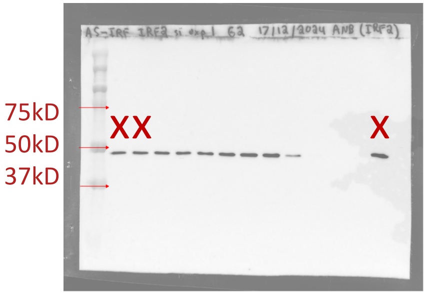

Cell line	A549
Treatments	IRF2 siRNA NS
Experiment # (Person)	Exp #1 (ANB)
Date of blot	17/12/2024
Person running blot	ANB
Primary Antibody	IRF2

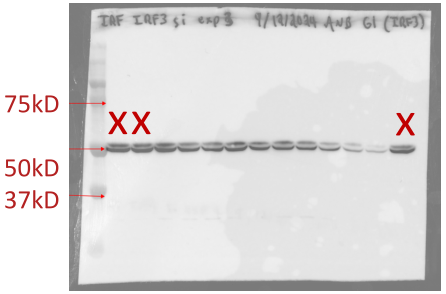

Cell line	A549
Treatments	IRF3 siRNA NS
Experiment # (Person)	Exp #3 (ANB)
Date of blot	9/12/2024
Person running blot	ANB
Primary Antibody	IRF3

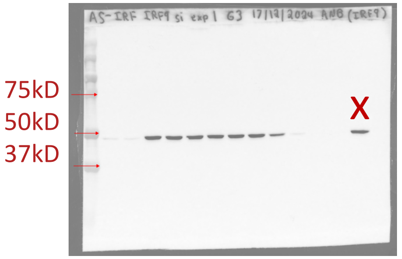

Cell line	A549
Treatments	IRF9 siRNA 24h IL-1β
Experiment # (Person)	Exp #1 (ANB)
Date of blot	17/12/2024
Person running blot	ANB
Primary Antibody	IRF9

GAPDH

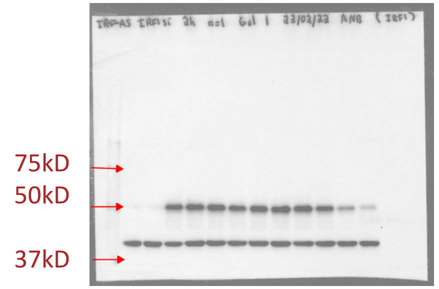

Cell line	A549
Treatments	IRF1 siRNA 2h IL1B
Experiment # (Person)	Exp #1 (ANB)
Date of blot	23/02/2022
Person running blot	ANB
Primary Antibody	GAPDH

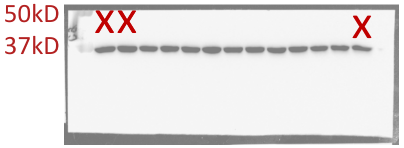

Cell line	A549
Treatments	IRF2 siRNA NS
Experiment # (Person)	Exp #1 (ANB)
Date of blot	18/12/2024
Person running blot	ANB
Primary Antibody	GAPDH

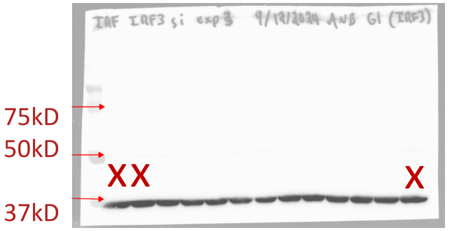

Cell line	A549
Treatments	IRF3 siRNA NS
Experiment # (Person)	Exp #3 (ANB)
Date of blot	10/12/2024
Person running blot	ANB
Primary Antibody	IRF3

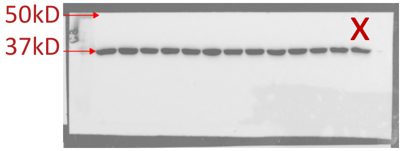

Cell line	A549
Treatments	IRF9 siRNA 24h IL-1β
Experiment # (Person)	Exp #1 (ANB)
Date of blot	17/12/2024
Person running blot	ANB
Primary Antibody	IRF9

Fig 2B

Loading order

IRF
CREB
GAPDH

IRF1

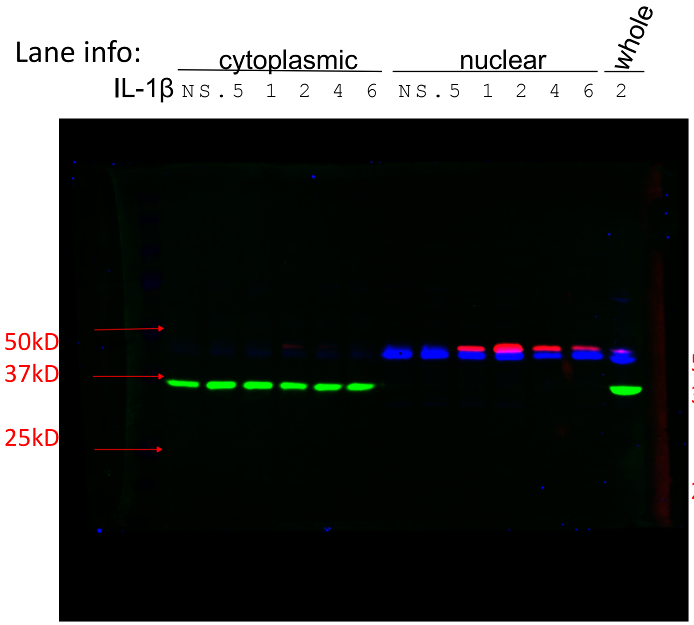

Cell line	A549
Treatments	Fractionation IL1B
Experiment # (Person)	Exp #4 (ANB)
Date of blot	02/2025
Person running blot	ANB
Primary Antibody	IRF1 + CREB + GAPDH

IRF2

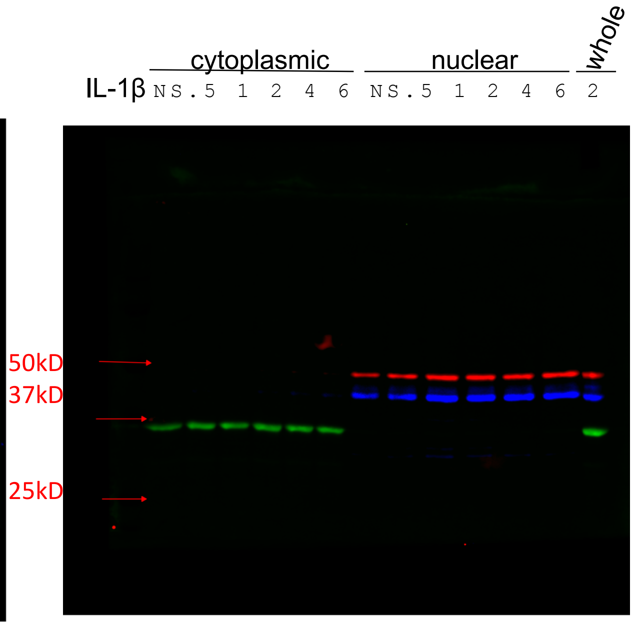

Cell line	A549
Treatments	Fractionation IL1B
Experiment # (Person)	Exp #5 (ANB)
Date of blot	02/2025
Person running blot	ANB
Primary Antibody	IRF2 + CREB + GAPDH

Fig 2B	IRF3	IRF9

Loading order

IRF
CREB
GAPDH

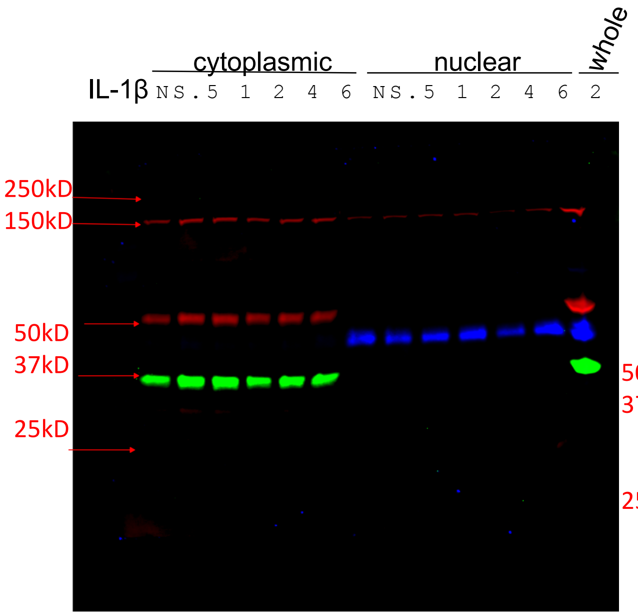

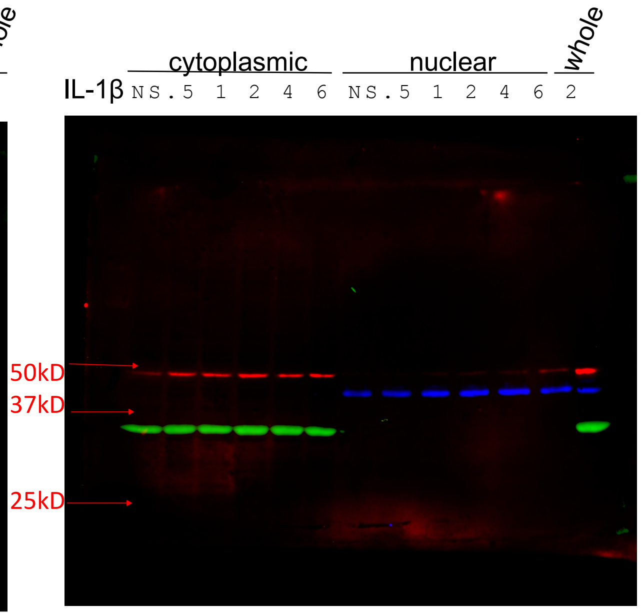

**Cell line**	**A549**
**Treatments**	Fractionation IL1B
**Experiment # (Person)**	Exp #4 (ANB)
**Date of blot**	02/2025
**Person running blot**	ANB
**Primary Antibody**	IRF3 + CREB + GAPDH

**Cell line**	**A549**
**Treatments**	Fractionation IL1B
**Experiment # (Person)**	Exp #5 (ANB)
**Date of blot**	02/2025
**Person running blot**	ANB
**Primary Antibody**	IRF9 + CREB + GAPDH

Fig 3A

Loading order

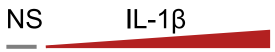

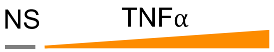

IRF

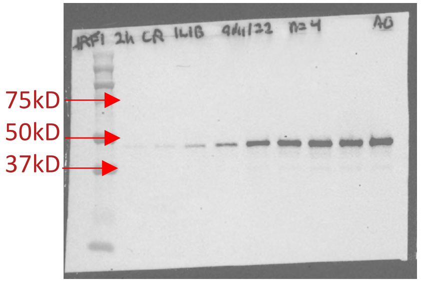

Cell line	A549
Treatments	IL-1β dose response
Experiment # (Person)	Exp #4 (AG)
Date of blot	9/11/22
Person running blot	AG
Primary Antibody	IRF1

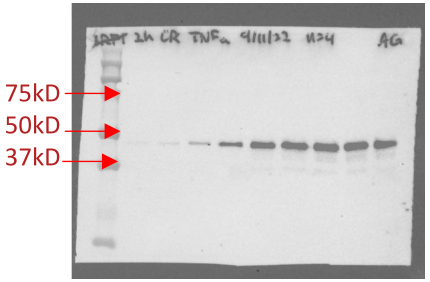

Cell line	A549
Treatments	TNFα dose response
Experiment # (Person)	Exp #4 (AG)
Date of blot	9/11/22
Person running blot	AG
Primary Antibody	IRF1

GAPDH

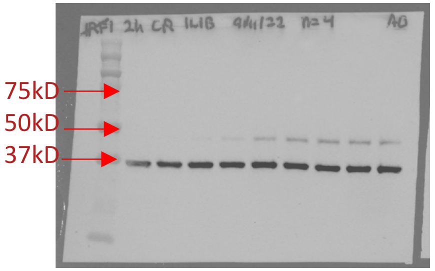

Cell line	A549
Treatments	IL-1β dose response
Experiment # (Person)	Exp #4 (AG)
Date of blot	10/11/22
Person running blot	AG
Primary Antibody	GAPDH

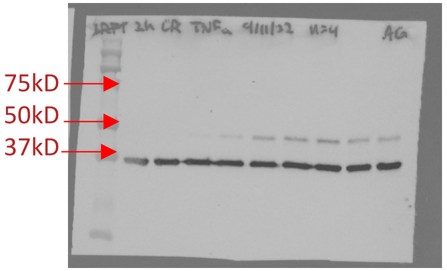

Cell line	A549
Treatments	TNFα dose response
Experiment # (Person)	Exp #4 (AG)
Date of blot	10/11/22
Person running blot	AG
Primary Antibody	GAPDH

Fig 3B

Loading order

IRF

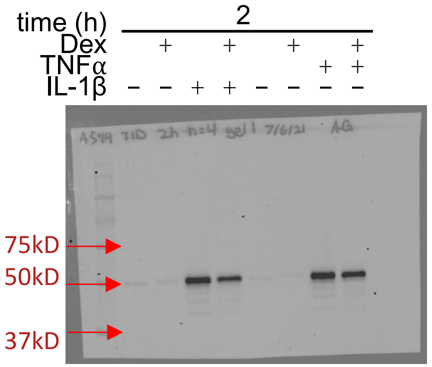

Cell line	A549
Treatments	TNFα/IL-1β/Dex 2h
Experiment # (Person)	Exp #4 (AG)
Date of blot	7/06/2021
Person running blot	AG
Primary Antibody	IRF1

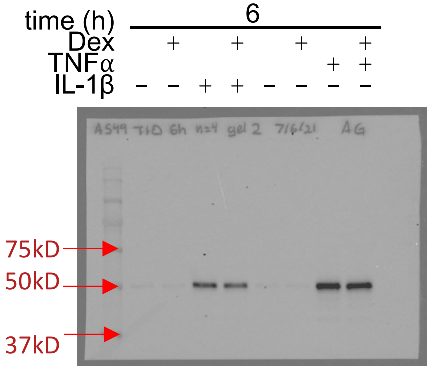

Cell line	A549
Treatments	TNFα/IL-1β/Dex 6h
Experiment # (Person)	Exp #4 (AG)
Date of blot	7/06/2021
Person running blot	AG
Primary Antibody	IRF1

GAPDH

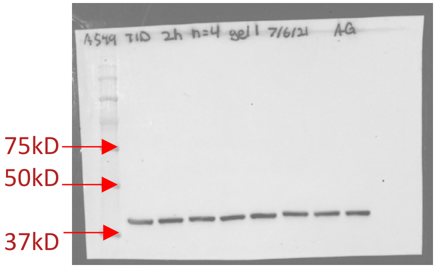

Cell line	A549
Treatments	TNFα/IL-1β/Dex 2h
Experiment # (Person)	Exp #4 (AG)
Date of blot	8/06/2021
Person running blot	AG
Primary Antibody	GAPDH

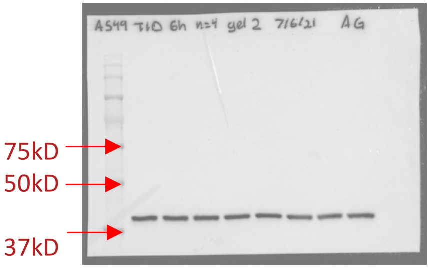

Cell line	A549
Treatments	TNFα/IL-1β/Dex 6h
Experiment # (Person)	Exp #4 (AG)
Date of blot	8/06/2021
Person running blot	AG
Primary Antibody	GAPDH

Fig 3C

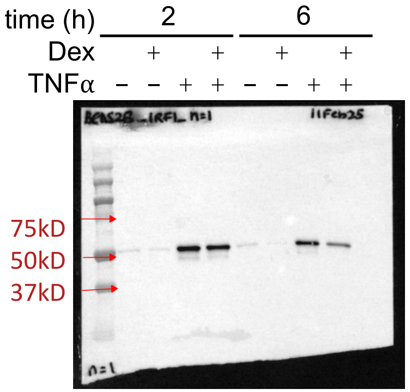

Cell line	BEAS2B
Treatments	TNFα//Dex 2h 6h
Experiment # (Person)	Exp #1 (PC)
Date of blot	11/02/2025
Person running blot	PC
Primary Antibody	IRF1

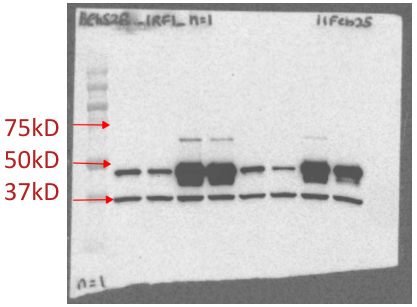

Cell line	BEAS2B
Treatments	TNFα//Dex 2h 6h
Experiment # (Person)	Exp #1 (PC)
Date of blot	
Person running blot	PC
Primary Antibody	IRF1

Fig 4A	IκBα	GFP	IRF1	GAPDH
Loading order				

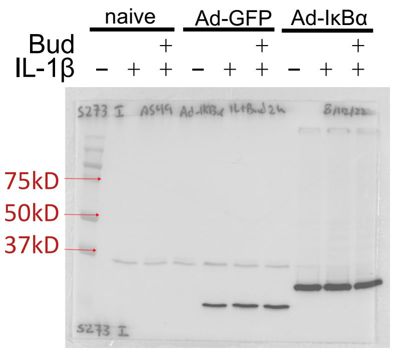

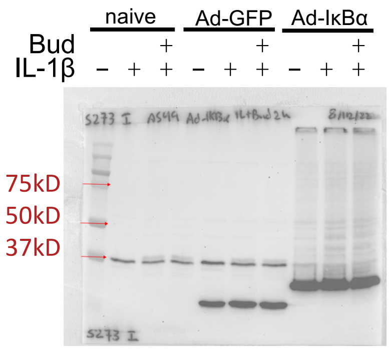

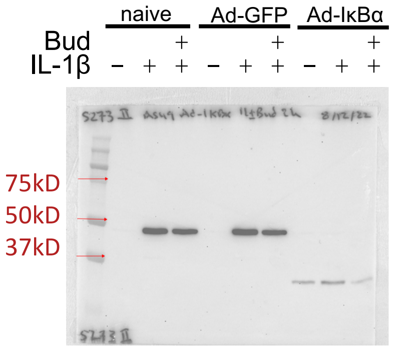

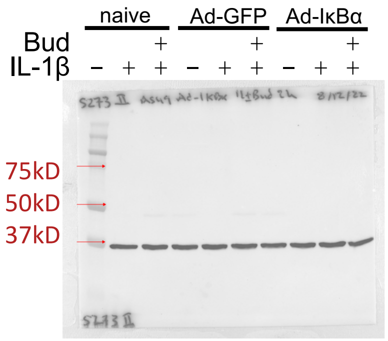

**Cell line**	**A549**
**Treatments**	IκBαΔN MOI 30 IL-1β
**Experiment # (Person)**	S273 (MM)
**Date of blot**	8/12/2022
**Person running blot**	MM
**Primary Antibody**	IκBα

**Cell line**	**A549**
**Treatments**	IκBαΔN MOI 30 IL-1β
**Experiment # (Person)**	S273 (MM)
**Date of blot**	8/12/2022
**Person running blot**	MM
**Primary Antibody**	GFP

**Cell line**	**A549**
**Treatments**	IκBαΔN MOI 30 IL-1β
**Experiment # (Person)**	S273 (MM)
**Date of blot**	8/12/2022
**Person running blot**	MM
**Primary Antibody**	IRF1

**Cell line**	**A549**
**Treatments**	IκBαΔN MOI 30 IL-1β
**Experiment # (Person)**	S273 (MM)
**Date of blot**	8/12/2022
**Person running blot**	MM
**Primary Antibody**	GAPDH

Fig 5A

p65

Loading order

p65 siRNA						+
C4 siRNA					+	
lipid		+		+		
IL-1β	–	–	+	+	+	+

IRF/ NF-κB

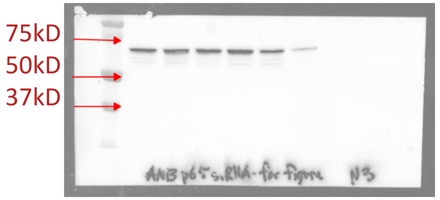

Cell line	A549
Treatments	p65 siRNA IL-1β
Experiment # (Person)	Exp 3 (KK AG)
Date of blot	2024
Person running blot	AG
Primary Antibody	IRF1

GAPDH

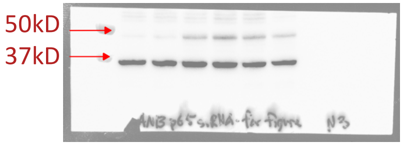

Cell line	A549
Treatments	p65 siRNA IL-1β
Experiment # (Person)	Exp 3 (KK AG)
Date of blot	2024
Person running blot	AG
Primary Antibody	GAPDH

IRF1

Loading order

p65 siRNA						+
C4 siRNA					+	
lipid		+		+		
IL-1β	–	–	+	+	+	+

IRF/ NF-κB

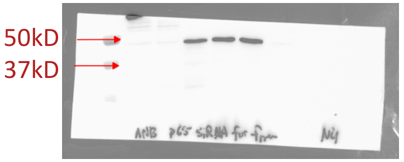

Cell line	A549
Treatments	p65 siRNA IL-1β
Experiment # (Person)	Exp 4 (KK AG)
Date of blot	2024
Person running blot	AG
Primary Antibody	p65

GAPDH

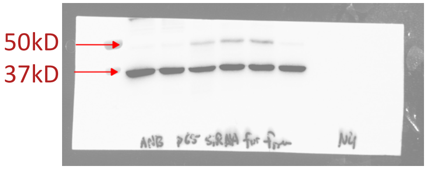

Cell line	A549
Treatments	p65 siRNA IL-1β
Experiment # (Person)	Exp 4 (KK AG)
Date of blot	2024
Person running blot	AG
Primary Antibody	GAPDH

Fig 6B

Loading order

	NS				IL-1β				TNFα			
PS-1145		+				+				+		
ML-120B			+				+				+	
TPCA-1	–	–	–	+	–	–	–	+	–	–	–	+

IRF

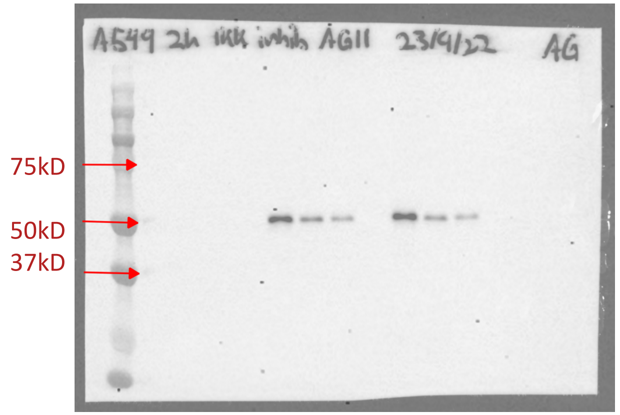

Cell line	A549
Treatments	IKK inhibs IL-1β TNFα
Experiment # (Person)	Exp #11 (AG)
Date of blot	23/09/2022
Person running blot	AG
Primary Antibody	IRF1

GAPDH

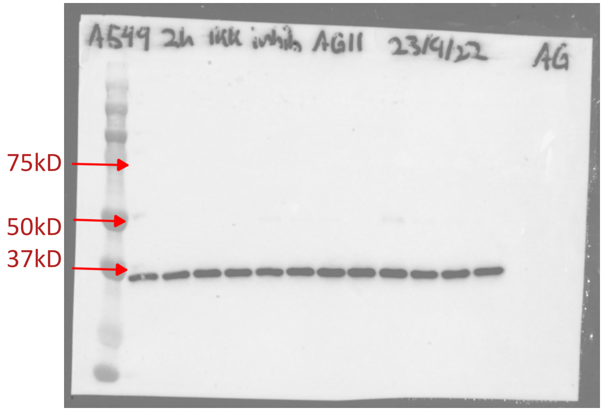

Cell line	A549
Treatments	IKK inhibs IL-1β TNFα
Experiment # (Person)	Exp #11 (AG)
Date of blot	24/09/2022
Person running blot	AG
Primary Antibody	GAPDH

Supplementary figures

Fig S2

Loading order

IRF1

time (h) 1 2 4 6 8 12 24
TNFα – + + + + + + + –

75kD
50kD
37kD

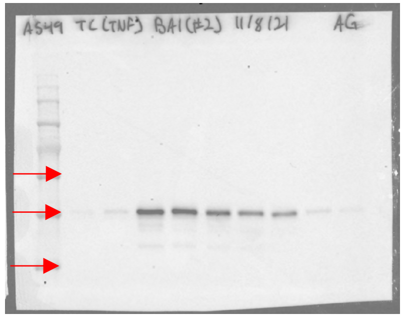

Cell line	A549
Treatments	TNFα TC
Experiment # (Person)	BA1 #2 (AG)
Date of blot	11/08/2021
Person running blot	AG
Primary Antibody	IRF1

IRF3

time (h) 1 2 4 6 8 12 24
TNFα – + + + + + + + –

75kD
50kD
37kD

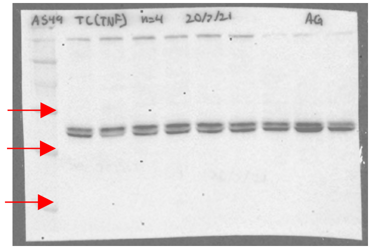

Cell line	A549
Treatments	TNFα TC
Experiment # (Person)	Exp 4 (AG)
Date of blot	20/27/2021
Person running blot	AG
Primary Antibody	IRF3

IRF

GAPDH

75kD
50kD
37kD

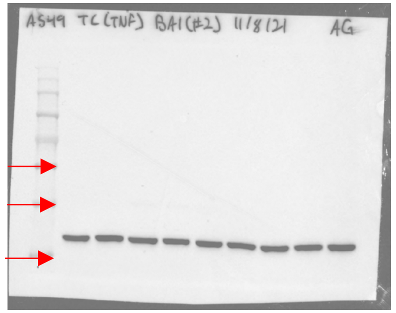

Cell line	A549
Treatments	TNFα TC
Experiment # (Person)	BA1 #2 (AG)
Date of blot	12/08/2021
Person running blot	AG
Primary Antibody	GAPDH

75kD
50kD
37kD

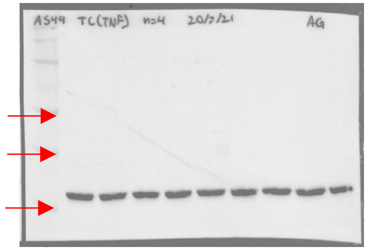

Cell line	A549
Treatments	TNFα TC
Experiment # (Person)	Exp 4 (AG)
Date of blot	20/27/2021
Person running blot	AG
Primary Antibody	GAPDH
