# Supplementary material for: Inflammatory cytokines promote interferon regulatory factor (IRF) transcriptional activity in human pulmonary epithelial cells through the induction of IRF1 by nuclear factor-κB
Source: PLoS One. 2025 Dec 8;20(12):e0329244. doi: 10.1371/journal.pone.0329244 (PMC12685198; doi:10.1371/journal.pone.0329244)
Supplement: Attachment — Submitted filename: Response to Reviewers PONE-D-25-37763.docx [file pone.0329244.s004.docx]

**Response to Reviewer comments**

PONE-D-25-37763

Inflammatory cytokines promote interferon regulatory factor (IRF) transcriptional activity in human pulmonary epithelial cells through the induction of IRF1 by nuclear factor-κB

PLOS ONE

Dear Dr. Newton,

Thank you for submitting your manuscript to PLOS ONE. After careful consideration, we feel that it has merit but does not fully meet PLOS ONE’s publication criteria as it currently stands. Therefore, we invite you to submit a revised version of the manuscript that addresses the points raised during the review process.

In your revised manuscript, please extensively work on the abstract. I recommend revising the abstract to improve its logical flow and thematic coherence. The current version is difficult to follow and unlikely to engage a broad scientific audience. As Reviewer #2 suggested, some of the data presentation needs to be reformatted and with suitable statistic analysis.

**Author response**: We have extensively reworked the abstract and feel this to now be much improved. We hope you and reviewers agree. Changes have been made to the graphs as per reviewer 2’s comment.

Please submit your revised manuscript by Oct 24 2025 11:59PM. If you will need more time than this to complete your revisions, please reply to this message or contact the journal office at [plosone@plos.org](mailto:plosone@plos.org). When you're ready to submit your revision, log on to <https://www.editorialmanager.com/pone/> and select the 'Submissions Needing Revision' folder to locate your manuscript file.

Please include the following items when submitting your revised manuscript:

- A rebuttal letter that responds to each point raised by the academic editor and reviewer(s). You should upload this letter as a separate file labeled 'Response to Reviewers'.
- A marked-up copy of your manuscript that highlights changes made to the original version. You should upload this as a separate file labeled 'Revised Manuscript with Track Changes'.
- An unmarked version of your revised paper without tracked changes. You should upload this as a separate file labeled 'Manuscript'.

If you would like to make changes to your financial disclosure, please include your updated statement in your cover letter. Guidelines for resubmitting your figure files are available below the reviewer comments at the end of this letter.

If applicable, we recommend that you deposit your laboratory protocols in protocols.io to enhance the reproducibility of your results. Protocols.io assigns your protocol its own identifier (DOI) so that it can be cited independently in the future. For instructions see: [https://journals.plos.org/plosone/s/submission-guidelines#loc-laboratory-protocols](https://can01.safelinks.protection.outlook.com/?url=https%3A%2F%2Ftrack.editorialmanager.com%2FCL0%2Fhttps%3A%252F%252Fjournals.plos.org%252Fplosone%252Fs%252Fsubmission-guidelines%2523loc-laboratory-protocols%2F1%2F010f0199307ce11e-e447b529-cd70-4b38-9dff-3d0531c69c81-000000%2Fd-QiRZ4feC2j0fJvXM0dYAGb8BW-V4y5-FqwplBl5ZI%3D227&data=05%7C02%7Crnewton%40ucalgary.ca%7C3d19ada7515f4343fc4c08ddefec0639%7Cc609a0eca5e346319686192280bd9151%7C1%7C0%7C638930519479815779%7CUnknown%7CTWFpbGZsb3d8eyJFbXB0eU1hcGkiOnRydWUsIlYiOiIwLjAuMDAwMCIsIlAiOiJXaW4zMiIsIkFOIjoiTWFpbCIsIldUIjoyfQ%3D%3D%7C0%7C%7C%7C&sdata=y2YXpuO747bYB%2BNF5PfrJPrE%2F0b95zsqL6S3ya%2BIHxQ%3D&reserved=0). Additionally, PLOS ONE offers an option for publishing peer-reviewed Lab Protocol articles, which describe protocols hosted on protocols.io. Read more information on sharing protocols at [https://plos.org/protocols?utm_medium=editorial-email&utm_source=authorletters&utm_campaign=protocols](https://can01.safelinks.protection.outlook.com/?url=https%3A%2F%2Ftrack.editorialmanager.com%2FCL0%2Fhttps%3A%252F%252Fplos.org%252Fprotocols%253Futm_medium%3Deditorial-email%2526utm_source%3Dauthorletters%2526utm_campaign%3Dprotocols%2F1%2F010f0199307ce11e-e447b529-cd70-4b38-9dff-3d0531c69c81-000000%2FhrKfCs_r5aNOARZOLV4b4CSOArCs00JSszLsKQkfg2U%3D227&data=05%7C02%7Crnewton%40ucalgary.ca%7C3d19ada7515f4343fc4c08ddefec0639%7Cc609a0eca5e346319686192280bd9151%7C1%7C0%7C638930519479882051%7CUnknown%7CTWFpbGZsb3d8eyJFbXB0eU1hcGkiOnRydWUsIlYiOiIwLjAuMDAwMCIsIlAiOiJXaW4zMiIsIkFOIjoiTWFpbCIsIldUIjoyfQ%3D%3D%7C0%7C%7C%7C&sdata=v60lzbGWUvygzKonkMtlauqNWW68ge2Jzw%2BPQoOUjFk%3D&reserved=0).

We look forward to receiving your revised manuscript.

Kind regards,

Helene Minyi Liu, Ph.D.
Academic Editor
PLOS ONE

Journal Requirements:

When submitting your revision, we need you to address these additional requirements.

1. Please ensure that your manuscript meets PLOS ONE's style requirements, including those for file naming. The PLOS ONE style templates can be found at

<https://journals.plos.org/plosone/s/file?id=wjVg/PLOSOne_formatting_sample_main_body.pdf> and

[https://journals.plos.org/plosone/s/file?id=ba62/PLOSOne_formatting_sample_title_authors_affiliations.pdf](https://can01.safelinks.protection.outlook.com/?url=https%3A%2F%2Ftrack.editorialmanager.com%2FCL0%2Fhttps%3A%252F%252Fjournals.plos.org%252Fplosone%252Fs%252Ffile%253Fid%3Dba62%252FPLOSOne_formatting_sample_title_authors_affiliations.pdf%2F1%2F010f0199307ce11e-e447b529-cd70-4b38-9dff-3d0531c69c81-000000%2FCiuuGqG1MrOLRksmg4ELcKQJAeaS9JiBlsG58L50h1c%3D227&data=05%7C02%7Crnewton%40ucalgary.ca%7C3d19ada7515f4343fc4c08ddefec0639%7Cc609a0eca5e346319686192280bd9151%7C1%7C0%7C638930519479936973%7CUnknown%7CTWFpbGZsb3d8eyJFbXB0eU1hcGkiOnRydWUsIlYiOiIwLjAuMDAwMCIsIlAiOiJXaW4zMiIsIkFOIjoiTWFpbCIsIldUIjoyfQ%3D%3D%7C0%7C%7C%7C&sdata=439w%2FUHdCUfUHM%2Be7oCjXTxB3I1je%2FLvz6YFWthSXB4%3D&reserved=0).

**Author response**: We have tried to adhere to all style and file naming requirements.

2. Please note that PLOS One has specific guidelines on code sharing for submissions in which author-generated code underpins the findings in the manuscript. In these cases, we expect all author-generated code to be made available without restrictions upon publication of the work. Please review our guidelines at <https://journals.plos.org/plosone/s/materials-and-software-sharing#loc-sharing-code> and ensure that your code is shared in a way that follows best practice and facilitates reproducibility and reuse.

**Author response**: There is no “code” underlying the findings in this manuscript. This said, various R studio packages were used to show data in various of the figures. This is noted in the relevant parts of the Materials and Methods.

3. Thank you for stating in your Funding Statement:

“This work was supported by: Grants from the Canadian Institutes of Health Research (CIHR) (funding reference numbers: PJT 156310 (RN) & 180480 (RN)) and Natural Sciences and Engineering Research Council of Canada (NSERC) discovery grants [RGPIN-2016-04549 & RGPIN-2023-03763] (RN); studentships (ANB): Alberta Graduate Excellence Scholarships (2020-2025).”

Please provide an amended statement that declares *all* the funding or sources of support (whether external or internal to your organization) received during this study, as detailed online in our guide for authors at <http://journals.plos.org/plosone/s/submit-now>.  Please also include the statement “There was no additional external funding received for this study.” in your updated Funding Statement.

Please include your amended Funding Statement within your cover letter. We will change the online submission form on your behalf.

**Author response**: The funding statement has been revised to “This work was supported by: Grants from the Canadian Institutes of Health Research (CIHR) (funding reference numbers: PJT 156310 (RN) & 180480 (RN)) and Natural Sciences and Engineering Research Council of Canada (NSERC) discovery grants [RGPIN-2016-04549 & RGPIN-2023-03763] (RN); studentships (ANB): Alberta Graduate Excellence Scholarships (2020-2025). The funders had no role in study design, data collection and analysis, decision to publish, or preparation of the manuscript. There was no additional external funding received for this study”

4. Thank you for stating the following financial disclosure:

“This work was supported by: Grants from the Canadian Institutes of Health Research (CIHR) (funding reference numbers: PJT 156310 (RN) & 180480 (RN)) and Natural Sciences and Engineering Research Council of Canada (NSERC) discovery grants [RGPIN-2016-04549 & RGPIN-2023-03763] (RN); studentships (ANB): Alberta Graduate Excellence Scholarships (2020-2025).”

Please state what role the funders took in the study.  If the funders had no role, please state: "The funders had no role in study design, data collection and analysis, decision to publish, or preparation of the manuscript."

If this statement is not correct you must amend it as needed.

Please include this amended Role of Funder statement in your cover letter; we will change the online submission form on your behalf.

**Author response**: As per above, the funding statement has been revised to “This work was supported by: Grants from the Canadian Institutes of Health Research (CIHR) (funding reference numbers: PJT 156310 (RN) & 180480 (RN)) and Natural Sciences and Engineering Research Council of Canada (NSERC) discovery grants [RGPIN-2016-04549 & RGPIN-2023-03763] (RN); studentships (ANB): Alberta Graduate Excellence Scholarships (2020-2025). The funders had no role in study design, data collection and analysis, decision to publish, or preparation of the manuscript. There was no additional external funding received for this study”.

We shall add this revised statement into the cover letter that supports this resubmission.

5. We noted in your submission details that a portion of your manuscript may have been presented or published elsewhere. [A549 and BEAS-2B ChIP-sequencing, and A549 RNA-sequencing datasets are published (BEAS-2B) or under review for publication (A549), but no specific data/graphs for IRFs used in this paper are included. All datasets are available in GEO.] Please clarify whether this [conference proceeding or publication] was peer-reviewed and formally published. If this work was previously peer-reviewed and published, in the cover letter please provide the reason that this work does not constitute dual publication and should be included in the current manuscript.

**Author response**: No part of this manuscript has been presented or published elsewhere. In this manuscript, we present new data in respect of the mRNA expression for the nine IRFs in human pulmonary epithelial cells. This has not been previously published. Likewise, we present ChIP-seq showing the recruitment of RELA, GR and RNA polymerase 2 (RNAP2) to the IRF1 gene locus. Again, these data have not been previously published. However, the mRNA sequencing are available under GEO Accession GSE295743 and the A549 ChIP sequencing data are available at GEO under accessions GSE296100 and GSE296101. These mRNA & ChIP sequencing datasets will be reported in a manuscript (“NF-κB-dependent redistribution of the GR cistrome recruits GR to inflammatory gene loci and correlates with reduced repression by glucocorticoid”) that is currently under review with iScience (ISCIENCE-D-25-07887R1). As per the statements above, none of the data concerning IRFs appears in this iScience manuscript, which this is more focused on describing overview patterns of behaviour. There is therefore no overlap between these two studies. The BEAS-2B ChIP sequencing data are available under GEO Accession GSE79803. Again, these data were previously described in overview terms in a publication from the Gerber lab (PMID: 27076634). However, there is no overlap, or duplication, in the current manuscript submission. Should you have any questions, or require further clarification, please do reach out.

6. We note that the grant information you provided in the ‘Funding Information’ and ‘Financial Disclosure’ sections do not match.

When you resubmit, please ensure that you provide the correct grant numbers for the awards you received for your study in the ‘Funding Information’ section.

**Author response**: These two statements have been modified to match each other and are also included in the cover letter.

7. Thank you for stating the following in the Acknowledgments Section of your manuscript:

“This work was supported by: Grants from the Canadian Institutes of Health Research (CIHR) (funding reference numbers: PJT 156310 (RN) & 180480 (RN)) and Natural Sciences and Engineering Research Council of Canada (NSERC) discovery grants [RGPIN-2016-04549 & RGPIN-2023-03763] (RN); studentships (ANB): Alberta Graduate Excellence Scholarships (2020-2025). All authors declare no competing interests with the contents of this article.”

We note that you have provided additional information within the Acknowledgements Section that is not currently declared in your Funding Statement. Please note that funding information should not appear in the Acknowledgments section or other areas of your manuscript. We will only publish funding information present in the Funding Statement section of the online submission form.

Please remove any funding-related text from the manuscript and let us know how you would like to update your Funding Statement. Currently, your Funding Statement reads as follows:

“This work was supported by: Grants from the Canadian Institutes of Health Research (CIHR) (funding reference numbers: PJT 156310 (RN) & 180480 (RN)) and Natural Sciences and Engineering Research Council of Canada (NSERC) discovery grants [RGPIN-2016-04549 & RGPIN-2023-03763] (RN); studentships (ANB): Alberta Graduate Excellence Scholarships (2020-2025).”

Please include your amended statements within your cover letter; we will change the online submission form on your behalf.

**Author response**: The funding statement has been removed from the text of the manuscript.

8. PLOS ONE now requires that authors provide the original uncropped and unadjusted images underlying all blot or gel results reported in a submission’s figures or Supporting Information files. This policy and the journal’s other requirements for blot/gel reporting and figure preparation are described in detail at <https://journals.plos.org/plosone/s/figures#loc-blot-and-gel-reporting-requirements> and <https://journals.plos.org/plosone/s/figures#loc-preparing-figures-from-image-files>. When you submit your revised manuscript, please ensure that your figures adhere fully to these guidelines and provide the original underlying images for all blot or gel data reported in your submission. See the following link for instructions on providing the original image data: <https://journals.plos.org/plosone/s/figures#loc-original-images-for-blots-and-gels>.

In your cover letter, please note whether your blot/gel image data are in Supporting Information or posted at a public data repository, provide the repository URL if relevant, and provide specific details as to which raw blot/gel images, if any, are not available. Email us at [plosone@plos.org](mailto:plosone@plos.org) if you have any questions.

**Author response**: The uncropped and unadjusted images for all the blots in the figures for this manuscript have been uploaded as “S2_raw_images.pdf”. We have also mentioned this in the covering letter.

9. We note that you have included the phrase “data not shown” in your manuscript. Unfortunately, this does not meet our data sharing requirements. PLOS does not permit references to inaccessible data. We require that authors provide all relevant data within the paper, Supporting Information files, or in an acceptable, public repository. Please add a citation to support this phrase or upload the data that corresponds with these findings to a stable repository (such as Figshare or Dryad) and provide and URLs, DOIs, or accession numbers that may be used to access these data. Or, if the data are not a core part of the research being presented in your study, we ask that you remove the phrase that refers to these data.

**Author response**: As per the request, the “data not shown” phrase is not necessary for the manuscript and has been removed in the revised version.

10. Your ethics statement should only appear in the Methods section of your manuscript. If your ethics statement is written in any section besides the Methods, please move it to the Methods section and delete it from any other section. Please ensure that your ethics statement is included in your manuscript, as the ethics statement entered into the online submission form will not be published alongside your manuscript.

**Author response**: We don’t not understand this request. The ethics statement was included in the methods section. Please let us know if there is something that we need to do.

11. Please include captions for your Supporting Information files at the end of your manuscript, and update any in-text citations to match accordingly. Please see our Supporting Information guidelines for more information: <http://journals.plos.org/plosone/s/supporting-information>.

**Author response**: Captions for the supplementary figures have been added at the end of the manuscript.

12.If the reviewer comments include a recommendation to cite specific previously published works, please review and evaluate these publications to determine whether they are relevant and should be cited. There is no requirement to cite these works unless the editor has indicated otherwise.

**Author response**: No such request was made.

13. Please review your reference list to ensure that it is complete and correct. If you have cited papers that have been retracted, please include the rationale for doing so in the manuscript text, or remove these references and replace them with relevant current references. Any changes to the reference list should be mentioned in the rebuttal letter that accompanies your revised manuscript. If you need to cite a retracted article, indicate the article’s retracted status in the References list and also include a citation and full reference for the retraction notice.

**Author response**: The reference list has been reviewed and contains no retracted papers.


[Note: HTML markup is below. Please do not edit.]

Reviewers' comments:

Reviewer's Responses to Questions

**Comments to the Author**

1. Is the manuscript technically sound, and do the data support the conclusions?

The manuscript must describe a technically sound piece of scientific research with data that supports the conclusions. Experiments must have been conducted rigorously, with appropriate controls, replication, and sample sizes. The conclusions must be drawn appropriately based on the data presented.

Reviewer #1: Yes

Reviewer #2: Yes

2. Has the statistical analysis been performed appropriately and rigorously?

Reviewer #1: Yes

Reviewer #2: Yes

3. Have the authors made all data underlying the findings in their manuscript fully available?

The [PLOS Data policy](https://can01.safelinks.protection.outlook.com/?url=http%3A%2F%2Ftrack.editorialmanager.com%2FCL0%2Fhttp%3A%252F%252Fwww.plosone.org%252Fstatic%252Fpolicies.action%2523sharing%2F1%2F010f0199307ce11e-e447b529-cd70-4b38-9dff-3d0531c69c81-000000%2FNZsiisQ_gRVwrgejE9cR-lrO0fyOi_ufEEp_runVaXo%3D227&data=05%7C02%7Crnewton%40ucalgary.ca%7C3d19ada7515f4343fc4c08ddefec0639%7Cc609a0eca5e346319686192280bd9151%7C1%7C0%7C638930519479995870%7CUnknown%7CTWFpbGZsb3d8eyJFbXB0eU1hcGkiOnRydWUsIlYiOiIwLjAuMDAwMCIsIlAiOiJXaW4zMiIsIkFOIjoiTWFpbCIsIldUIjoyfQ%3D%3D%7C0%7C%7C%7C&sdata=SIqBe6yyzEKfqFTK2r8Q%2B8%2BsICbLKMrrvu%2FPg9ZjUQw%3D&reserved=0) requires authors to make all data underlying the findings described in their manuscript fully available without restriction, with rare exception (please refer to the Data Availability Statement in the manuscript PDF file). The data should be provided as part of the manuscript or its supporting information, or deposited to a public repository. For example, in addition to summary statistics, the data points behind means, medians and variance measures should be available. If there are restrictions on publicly sharing data—e.g. participant privacy or use of data from a third party—those must be specified.

Reviewer #1: Yes

Reviewer #2: Yes

4. Is the manuscript presented in an intelligible fashion and written in standard English?

PLOS ONE does not copyedit accepted manuscripts, so the language in submitted articles must be clear, correct, and unambiguous. Any typographical or grammatical errors should be corrected at revision, so please note any specific errors here.

Reviewer #1: Yes

Reviewer #2: Yes

5. Review Comments to the Author

Please use the space provided to explain your answers to the questions above. You may also include additional comments for the author, including concerns about dual publication, research ethics, or publication ethics. (Please upload your review as an attachment if it exceeds 20,000 characters)

Reviewer #1: This manuscript examines the regulation of IRFs in human pulmonary epithelial cells, with a particular emphasis on the NFκB–dependent induction of IRF1 by IL1β and TNFα. The authors use a combination of cell lines (A549 and BEAS-2B) and primary human bronchial epithelial cells, applying multiple complementary approaches, including mRNA-seq, ChIP-seq, siRNA silencing, pharmacologic inhibition, and reporter assays, to dissect the underlying mechanisms.

The data convincingly demonstrate that IRF1 is robustly induced in an NFκB-dependent manner, with several upstream enhancer regions contributing to its transcriptional control. Notably, IRF1 induction appears largely resistant to glucocorticoid suppression, a finding with potential implications for glucocorticoid resistant airway inflammation and severe asthma.

Overall, this is a technically rigorous, well controlled, and carefully executed study that makes an original contribution to the field. The conclusions are well supported by the data, the manuscript is clearly written, and all required ethics approvals and data availability statements are in place.

I have only one minor suggestion:

The abstract is difficult to follow in its current form. It should be revised for clarity and improved flow so that the key findings are more easily understood.

**Author response**: Many thanks for the above comments. In the revised submission, the abstract has been extensively revised, we hope that this makes the key findings clearer. Certainly, we feel that this structure is preferred over the prior version.

Reviewer #2: During inflammation, cytokines such as IL-1β and TNF⍺ activate signaling cascades through their respective receptors, leading to the activation of transcription factors like NF-κB and interferon regulatory factors (IRFs). NF-κB is activated through the phosphorylation and degradation of IκBα, allowing NF-κB to translocate to the nucleus and drive inflammatory gene expression. Although IRFs are primarily known for their role in antiviral and interferon responses, emerging evidence suggests they may also be activated through NF-κB-associated pathways, though this remains underexplored. Airway epithelial cells (AECs), as the frontline defenders against inhaled pathogens and irritants, play a central role in lung inflammation and are key targets of inhaled glucocorticoids used to manage asthma. However, the regulation of IRFs—particularly IRF1—in AECs is not well understood. IRF1 is inducible by cytokines and may regulate inflammatory mediators such as CXCL10, which has been linked to severe asthma. This study investigates how IL-1β and TNF⍺ influence IRF1 expression and inflammatory signaling in AECs, and how these responses are modulated by glucocorticoids like dexamethasone and budesonide. There are some comments as the follows:

1. In some graphs, such as Fig 1A, Fig 10A, Supplementary Fig 1C, and Supplementary Fig 6A, the time intervals in the x-axis are different, so they are not suitable for line chart.

**Author response**: Each of these figures (Fig 1A, 10A, S1C, S6A) have been amended to have a “natural” x axis (i.e. time). Frankly, we think this is much clearer – thank you for this suggestion.

2. In Fig 3B and 3C, Fig 4, Fig 6A and Fig 9, the statistical results of some charts need to be fully labeled and presented. For example, in Fig 3, some are labeled with “ns”, but it’s confused whether non-labeled are all “ns”.

**Author response**: We apologize for this. We agree with the comment as raised and in the revised manuscript, we have removed all of the “ns” labels from the graphs. Only the labelled comparisons are significant.

3. In Fig 3 and Fig 9, should TNF⍺ treatment groups be labeled as yellow or orange?

**Author response**: In Figures 3 and 9, the TNF group is labelled in a yellowy-orange colour (colour code #E69F00) throughout the entire manuscript. This is to distinguish it from the IL-1β treatment that is always labelled in red. We can modify the colouring if the reviewer would prefer.

4. In line 99, “This revealed transcripts for all nine IRFs under basal conditions…." should be “These revealed transcripts……".

**Author response**: We agree that this wording was unclear. In the revised manuscript this line has been amended to say “This analysis revealed transcripts for all nine IRFs under basal conditions.”

6. PLOS authors have the option to publish the peer review history of their article ([what does this mean?](https://can01.safelinks.protection.outlook.com/?url=https%3A%2F%2Ftrack.editorialmanager.com%2FCL0%2Fhttps%3A%252F%252Fjournals.plos.org%252Fplosone%252Fs%252Feditorial-and-peer-review-process%2523loc-peer-review-history%2F1%2F010f0199307ce11e-e447b529-cd70-4b38-9dff-3d0531c69c81-000000%2FCq3zrDyj4aIGj6pWr565O5CNQRJ-nmOcW_rsQwF5RPA%3D227&data=05%7C02%7Crnewton%40ucalgary.ca%7C3d19ada7515f4343fc4c08ddefec0639%7Cc609a0eca5e346319686192280bd9151%7C1%7C0%7C638930519480051125%7CUnknown%7CTWFpbGZsb3d8eyJFbXB0eU1hcGkiOnRydWUsIlYiOiIwLjAuMDAwMCIsIlAiOiJXaW4zMiIsIkFOIjoiTWFpbCIsIldUIjoyfQ%3D%3D%7C0%7C%7C%7C&sdata=7ZJQa3Ff%2F43Zlv3I8Bg4jKXA%2F3tLkwnZo%2FFq8%2FpDnKI%3D&reserved=0)). If published, this will include your full peer review and any attached files.


If you choose “no”, your identity will remain anonymous but your review may still be made public.


**Do you want your identity to be public for this peer review?** For information about this choice, including consent withdrawal, please see our [Privacy Policy](https://can01.safelinks.protection.outlook.com/?url=https%3A%2F%2Ftrack.editorialmanager.com%2FCL0%2Fhttps%3A%252F%252Fwww.plos.org%252Fprivacy-policy%2F1%2F010f0199307ce11e-e447b529-cd70-4b38-9dff-3d0531c69c81-000000%2FOKw9PXaoFSJ79EPjqQpRj_8-xUOeiQGEx46ke3f_wXA%3D227&data=05%7C02%7Crnewton%40ucalgary.ca%7C3d19ada7515f4343fc4c08ddefec0639%7Cc609a0eca5e346319686192280bd9151%7C1%7C0%7C638930519480110851%7CUnknown%7CTWFpbGZsb3d8eyJFbXB0eU1hcGkiOnRydWUsIlYiOiIwLjAuMDAwMCIsIlAiOiJXaW4zMiIsIkFOIjoiTWFpbCIsIldUIjoyfQ%3D%3D%7C0%7C%7C%7C&sdata=HPfuAxLYSbYukQ%2FgKG27z9bq0dQn89o8ZGlBPgD1H74%3D&reserved=0).

Reviewer #1: No

Reviewer #2: No
